# Association of Low Fecal Elastase-1 and Non-Ulcer Dyspepsia

**DOI:** 10.3390/jcm7060155

**Published:** 2018-06-16

**Authors:** Mustafa Tahtaci, Huseyin Koseoglu, Murat Alisik, Oyku Tayfur Yurekli, Gozde Tahtaci, Ozcan Erel, Osman Ersoy

**Affiliations:** 1 Department of Gastroenterology, Faculty of Medicine, Yıldırım Beyazıt University, Ankara 06800, Turkey; oykutayfur78@yahoo.com (O.T.Y.); oersoy@yahoo.com.tr (O.E.); 2 Department of Gastroenterology, Faculty of Medicine, Hitit University, Corum 19040, Turkey; huseyinko@yahoo.com; 3 Department of Biochemistry, Faculty of Medicine, Yıldırım Beyazıt University, Ankara 06800, Turkey; muratalisik@gmail.com (M.A.); erelozcan@gmail.com (O.E.); 4 Department of Internal Medicine, Faculty of Medicine, Gazi University, Ankara 06560, Turkey; drgozdetahtaci@gmail.com

**Keywords:** exocrine pancreas, dyspepsia, endoscopy

## Abstract

Non-ulcer dyspepsia (NUD) is a term used to define a set of symptoms that are believed to originate from the gastroduodenal region, and no underlying organic, systemic, or metabolic reason can be found. The majority of patients suffer from chronic symptoms although half of the patients report improvement in symptoms with time. The potential role exocrine pancreatic insufficiency in NUD patients has not been clarified yet. We aimed to identify exocrine pancreas function with pancreatic fecal elastase-1 in patients diagnosed with non-ulcer dyspepsia and no typical exocrine pancreatic insufficiency (EPI) symptoms. Thirty-five patients referred to gastroenterology clinics with NUD and 35 people with no dyspeptic symptoms as a control group were included in this prospective study. Non-ulcer dyspepsia patients were classified as group 1 and control subjects classified as group 2. Upper gastrointestinal endoscopies were performed in both groups. Assessment of exocrine pancreatic function was performed by measuring fecal elastase-1 concentration with a commercial ELISA kit using polyclonal antibodies (BioServ Diagnostics) in NUD patients compared to control subjects. Mean fecal elastase-1 levels were significantly lower in group 1 patients compared with group 2 (367.47 ± 43.27; 502.48 ± 50.94 respectively; *p* = 0.04). The percentage of the patients with EPI was significantly higher in group 1 (*p* = 0.02). Patients with NUD should be re-evaluated if they do not show satisfactory improvement with treatment. Exocrine pancreatic insufficiency was significantly higher in patients with NUD in our study. Evaluation for the presence of EPI can be a cost effective approach in management of refractory patients during the process of ruling out organic reasons.

## 1. Introduction

Dyspepsia can be basically defined as a feeling of discomfort in the upper abdominal region. Non-ulcer (or functional) dyspepsia (NUD) is a general term used to define chronic dyspepsia [1]. Dyspepsia can be classified into 2 groups, i.e., non-ulcer and organic [2]. Non-ulcer dyspepsia is a term used to define a set of symptoms that are believed to originate from gastroduodenal region, and no underlying organic, systemic, or metabolic reason can be found [3]. In only 40% of dyspepsia patients, an underlying organic reason can be found [4]. Diagnosis of NUD can be put forward after ruling out organic reasons. NUD patients are significant sources of health expenditures and work productivity is significantly lost in these patients [5].

Exocrine pancreatic insufficiency (EPI) can be defined as the failure of normal digestion due to exocrine pancreas enzyme deficiency. Steatorrhea and weight loss typically accompany EPI [6]. The relationship between the pancreas and dyspepsia was defined in 1949 as “pancreatic dyspepsia” [7]. Pancreas dysfunction and pancreatic diseases were proposed to relate with gastrointestinal diseases [8]. In patients with dyspepsia, due to unidentified reasons, serum lipase levels were found to be elevated and it was proposed that mild non-ulcer pancreatic insufficiency could be present in these patients [9]. Endosonography can reveal abnormalities in pancreas in patients with persistent dyspepsia [10]. In a study evaluating serum activity of pancreatic isoamylase, dyspepsia was found to be a common symptom in patients with pancreatic diseases [11]. Pancreatitis and pancreas cancer are defined as rare causative factors for dyspepsia etiology. Nevertheless no recommendation is given so far for EPI screening [12].

As non-ulcer dyspepsia is a common health problem with mostly unsatisfactory treatment results, should EPI be ruled out before diagnosis? We aimed to identify exocrine pancreas function with pancreatic fecal elastase-1 in patients diagnosed with NUD and no typical EPI symptoms.

## 2. Materials and Methods

### 2.1. Study Population and Exclusion Criteria

The study was conducted between 2015 and 2016 in Ankara Ataturk training and Research hospital, which is a tertiary referral center in Turkey. Thirty-five patients from the gastroenterology polyclinics, who are diagnosed as having non-ulcer dyspepsia and 35 age and gender matched controls without dyspepsia, were prospectively recruited for the study. Patients who had taken a proton pump inhibitor for a defined period without satisfactory symptomatic response were chosen as non-ulcer dyspepsia patients. Non-ulcer dyspepsia patients and control subjects were classified as NUD and control groups, respectively. Patients with symptoms of steatorrhea, chronic diarrhea, chronic pancreatitis, history of regular alcohol intake and past history of celiac disease, patients with a macroscopic endoscopic findings suggesting diseases that may cause malabsorption like celiac disease, inflammatory bowel disease, cystic fibrosis, and gastric or pancreatic surgery were excluded from the study. Also patients with watery stool samples were not included in the study. Ethics committee approval was received for this study from the ethics committee of Yildirim Beyazit University Faculty of Medicine (Date: 12 November 2014, Approval Number: 186). Written informed consent was obtained from patients who participated in this study.

### 2.2. Endoscopic Examination and Diagnosis of NUD

Upper gastrointestinal endoscopies were performed with GIF-H180J, Olympus, Japan in both patients and the control group. In the control group, upper gastrointestinal endoscopies were performed for reasons other than dyspepsia. Reasons of referral were pyrosis, dysphagia, investigation of the etiology of anemia, or evaluation of portal hypertension with decreasing incidence. Non-ulcer dyspepsia was diagnosed in patients complaining about pain or discomfort in epigastric region lasting for at least 1 year with no ulcers detected upon endoscopy. Non-ulcer dyspepsia patients are further classified into 2 categories as epigastric pain syndrome and postprandial distress syndrome, according to dominant complaints [13]. Non-ulcer dyspepsia patients were evaluated for the presence of Helicobacter Pylori (HP) with a rapid urease test of antral biopsies. Biopsies were not taken from patients with the past history of evaluation for the presence of HP with negative results.

### 2.3. Fecal Elastase-1 Measurements and Diagnosis of EPI

Stool samples with solid consistency were collected and immediately stored at −80 °C until analyzing time. Fecal elastase concentrations of all samples were measured at the same time with commercially available enzyme linked immunosorbent assay (ELISA) kit (BIOSERVE Diagnostics GmbH, Rostock, Germany) according to the manufacturer’s instructions. Measurements were defined as Fecal Elastase-1/gram stool. Fecal Elastase-1 levels > 200 μg/g were defined as normal, Fecal Elastase-1 levels < 100 μg/g were defined as EPI [14].

### 2.4. Statistical Analyses

Preliminary analyses were completed for frequencies, means, standard deviation (SD), and percentages, where applicable. Categorical variables were compared using the chi-square test or Fisher’s exact test as appropriate. Normality of the distribution of the continuous variables was evaluated using the Shapiro-Wilk’s test. Continuous variables were compared by independent *t*-tests or Mann–Whitney *U*. All statistical analysis was performed using SPSS version 17.0 (SPSS, Chicago, IL, USA).

## 3. Results

### 3.1. Characteristics of Patients

There were no statistically significant differences in terms of male/female ratios and age (*p* = 0.80 and *p* = 0.85, respectively). There were more patients with diabetes in NUD group but this difference did not reach to statistical significance. (*p* = 0.39). There were no statistically significant differences in terms of mean hemoglobin, fasting plasma glucose, ALT, AST, amylase and lipase levels between groups 1 and 2. (*p* = 0.47, *p* = 0.21, *p* = 0.60, *p* = 0.68, *p* = 0.85, *p* = 0.64, respectively) (Table 1). In NUD the group ratios of postprandial Distress Syndrome and Epigastric Pain Syndrome were not statistically different (57.1% vs. 42.9%, *p* = 0.170).

### 3.2. Fecal Elastase-1 Measurements

Mean fecal elastase-1 levels were statistically lower in NUD patients. (367.47 ± 43, 502.48 ± 50, respectively, *p* = 0.04). The percentage of patients with EPI were significantly higher in the NUD group. (*p* = 0.02). (Table 1). Mean age of patients with EPI was 64.8 ± 12.6 years. The percentage of patients with postprandial Distress Syndrome and Epigastric Pain Syndrome was not different in NUD patients with EPI (60% vs. 40%, respectively *p* = 0.50). Presence of HP positivity or negativity was not different in NUD patients with EPI (40% vs. 60%, respectively *p* = 0.50).

## 4. Discussion

Despite extensive research for the determination of the reason of NUD in the last decade, exact pathophysiology could not be elucidated. Various theories have been formulated to identify underlying pathophysiology in NUD but none of them were proven yet [15]. This study shows that EPI can be present in some NUD patients.

Non-ulcer dyspepsia is a common health problem with prevalences ranging between 10% to 30% [16]. Endoscopic evaluation is one of the most important tools to exclude an organic pathology [17]. Patients with dyspepsia are frequently evaluated with endoscopy and patients with no ulcers upon endoscopy are accepted as having NUD.

In a study which recruited dyspeptic patients, consecutively chronic pancreatitis and pancreas cancer were found as rare causes of dyspepsia [18]. On the contrary, Gullo, et al. reported that dyspeptic symptoms, other than recurrent abdominal pain, were rarely found in chronic pancreatitis [19]. On the other hand, in another study, which evaluated dyspeptic patients, 35% of the patients were found to have diminished pancreatic function [20]. Those conflicting reports could have resulted from the diagnosis of dyspepsia only with symptoms and problems in exclusion criteria. We found EPI in 14.3% of our NUD patients, a percentage higher than expected in NUD patients. Our inclusion criteria were more strict than most of the studies in the literature. We included patients with complaints for at least 1 year with no ulcer on endoscopy. Also, we excluded patients with steatorrhea, chronic diarrhea, chronic pancreatitis, history of regular alcohol intake and past history of celiac disease, inflammatory bowel disease, cystic fibrosis, and gastric or pancreatic surgery, which may change fecal elastase-1 levels. These rather selective study protocols may have caused lower EPI ratios compared to the study described previously [20].

NUD patients were classified into epigastric pain syndrome and postprandial distress syndrome. Although epigastric pain syndrome was more common in NUD patients there were no significant differences regarding symptoms. Furthermore in NUD patients with EPI there were no significant differences in terms of symptoms. Proposed pathophysiological mechanisms for the symptoms in NUD have been defined as delayed gastric emptying, impaired gastric accommodation to a meal, hypersensitivity to gastric distention, HP infection, altered duodenal response to lipids or acid, altered antroduodenojejunal motility, abnormalities of gastric electrical rhythm, unsuppressed postprandial phasic contractility in the proximal stomach, and autonomic and central nervous system dysregulation [21]. Helicobacter Pylori infection may play a role in the pathogenesis of acute and chronic pancreatitis [22]. We did not find any significant difference in patients with EPI in terms of HP positivity.

The pancreas may act in various steps in NUD pathogenesis. Exocrine pancreatic insufficiency can itself cause motility disorders or exacerbate underlying motility disorders [23]. In a study evaluating altered vagal and intestinal mechanosensory function in patients with chronic unidentified dyspepsia, the pancreatic polypeptide response to insulin induced hypoglycemia had been shown to diminished [24]. The same study also showed that efferent vagal function had been impaired in patients with chronic unidentified dyspepsia [24]. In a study evaluating 22 endoscopy proven NUD patients, low levels of mean tryptic activity had been proposed to explain the symptoms in some patients. Chemosensors, which are important in the regulation of physiological homeostasis mechanisms via producing neurohumoral responses, had been proposed to play a role in NUD [25]. Lariño-Noia, J., et al. defined frequent morphological and functional changes in chronic pancreatitis patients with epigastric pain syndrome like symptoms. Nevertheless they suggested that more research had been needed to define whether these pancreatic changes could explain the presentation of epigastric pain syndrome-like symptoms [26].

Although EPI has been thought to be important in the pathophysiology of dyspepsia, effect of pancreatic enzyme replacement on symptoms show significant differences. In a study done by Kleveland, P.M., et al. short term pancreatic enzyme replacement failed to improve symptoms in NUD patients [27]. On the other hand, gastric myoelectricity had been shown to be impaired in chronic pancreatitis and improved with pancreatic enzyme replacement [28].

## 5. Conclusions

Exocrine pancreatic insufficiency can accompany NUD. When standard laboratory evaluation cannot reveal the reason of dyspepsia, investigation of EPI can be an appropriate strategy. Clearly more studies with larger patient numbers are needed to evaluate the effect of pancreatic enzyme replacement on symptoms.

## 6. Limitations

Patients with EPI were evaluated with abdominal ultrasonography to define any underlying pancreas pathology and no cases of chronic pancreatitis or pancreas cancer had been detected. But as a limitation, these patients were not evaluated using advanced imaging techniques like endosonography. Another limitation is that we did not evaluate gastric emptying time in patients with EPI. Another limitation is that although the prevalence of EPI was high in EPI patients, a small number of patients prevents us from reaching firm conclusions. More studies with bigger sample sizes are needed to support our findings. One of the main limitations of the current study is that we did not evaluate the symptomatic response to enzyme replacement therapy. The other limitation of our study is the lack of desired diagnostic performance of fecal elastase in the diagnosis of EPI [29]. The application of direct pancreatic function tests, instead of an indirect test like FE-1, may be more suitable for research purposes. Finally, a lack of objective exclusion of some disease processes like chronic pancreatitis may represent another limitation of our study. We believe further studies should better be performed in this area.

## Figures and Tables

**Table 1:** Comparison of demographic, clinical and laboratory data of NUD patients and the control group.

	NUD (*n* = 35)	Controls (*n* = 35)	*p*-Value
**Female/male**	20/15	22/13	0.80
**Age (years)**	53.77 ± 2.47	53.11 ± 2.68	0.85
**Diabetes *n* (%)**	10 (28.6)	6 (17.1)	0.39
**Hemoglobine**	14.2 ± 1.17	13.95 ± 0.27	0.47
**FPG (mg/dL)**	99.51 ± 3.51	93.65 ± 3.05	0.21
**ALT (IU/L)**	20.82 ± 1.47	22.65 ± 3.19	0.60
**AST (IU/L)**	20.54 ± 1.18	21.65 ± 2.43	0.68
**Amylase (IU/L)**	73.57 ± 4.02	74.57 ± 3.67	0.85
**Lipase (IU/L)**	32.02 ± 1.77	33.22 ± 1.89	0.64
**Fecal elastase-1 (μg/g)**	367.47 ± 43.27	502.48 ± 50.94	* 0.04
**EPI *n* (%)**	5(14.3)	0(0)	* 0.02

FPG: fasting plasma glucose; AST: Aspartate transaminotransferase; ALT: Alanine transaminotransferase; EPI: Exocrine pancreatic insufficiency. Parameters were expressed as *n* (%) or mean with standard error. chi-square test, Fisher’s exact test, independent *t*-tests, Mann–Whitney U test * Statistically significant.
